# The Impact of Opium Abuse on Lipid Profile in Patients with Diabetes: A Systematic Review and Meta-Analysis

**DOI:** 10.3390/ijerph16234795

**Published:** 2019-11-29

**Authors:** Omorogieva Ojo, Xiao-Hua Wang, Osarhumwese Osaretin Ojo, Jude Ibe

**Affiliations:** 1 School of Health Sciences, University of Greenwich, London SE9 2UG, UK; 2 The School of Nursing, Soochow University, Suzhou 215006, China; wangxiaohua@suda.edu.cn; 3 South London and Maudsley NHS Foundation Trust, University Hospital, Lewisham High Street, London SE13 6LH, UK; Osarhumwese.Ojo@slam.nhs.uk; 4 School of Health Sciences, University of Greenwich, London SE9 2UG, UK; J.C.Ibe@greenwich.ac.uk

**Keywords:** diabetes, substance abuse, opioids, total cholesterol, HDL cholesterol, LDL cholesterol, triglyceride, meta-analysis, systematic review, body mass index

## Abstract

There is an increasing prevalence of diabetes worldwide and substance abuse has been observed as a problem among some people with diabetes. Therefore, there is an urgent need to understand the association between unhealthy drug use including the abuse of opium and clinical outcomes including its impact on lipid profile in patients with diabetes as the presence of these conditions can increase the risk of cardiovascular morbidity and mortality. Aim: This was a systematic review and meta-analysis which evaluated the impact of opium abuse on lipid profile in patients with diabetes. Method: This systematic review was conducted in line with the preferred reporting items for systematic reviews and meta-analyses (PRISMA) guidelines. Three databases (Embase, PubMed, and PsycINFO) plus Google Scholar were searched for relevant articles from database inception to 18 July 2019 based on the Population, Intervention, Comparator, and Outcomes (PICO) framework. The studies included were based on a set of inclusion and exclusion criteria including patients with diabetes who abused opium. Articles were evaluated for risk of bias and the meta-analysis was conducted using Revman. Results: Six articles that met the criteria were included in the systematic review and meta-analysis. The type of substance abused was opium in all the studies. The results of the meta-analysis showed that opium abuse significantly (*P* = 0.01) lowered total cholesterol compared to control with a mean difference of −0.17 (95% CI, −0.29, −0.04) in patients with diabetes. With respect to high-density lipoprotein (HDL) cholesterol, low-density lipoprotein (LDL) cholesterol, triglycerides, and body mass index, the differences were not statistically significant (*P* > 0.05) between those who abused opium compared with the control. Nutritional deficiencies, weight loss and lipid dysregulation due to liver dysfunction which are found in people who abuse substances may explain the findings of the current review with respect to lipid profile in patients with diabetes who abuse opium compared with the control. Conclusion: The findings of this systematic review and meta-analysis have shown that opium abuse significantly decreased total cholesterol (*P* < 0.05) in patients with diabetes. However, the effect of opium abuse on HDL cholesterol, triglycerides, body mass index (BMI) and LDL cholesterol in these patients were not statistically significant (*P* > 0.05) compared with the control. This result has public health significance in terms of ensuring the promotion of adequate nutritional intake in patients with diabetes who abuse opium.

## 1. Introduction

There is an increasing prevalence of diabetes worldwide and this presents a significant public health challenge [1,2]. In 2014, there were 422 million people who were living with diabetes compared to 108 million people in 1980, representing a global increase in the prevalence of diabetes from 4.7% in 1980 to 8.5% in 2014 among the adult population [1]. In addition, diabetes is projected to be the seventh leading cause of death by 2030 [3]. There is evidence of increased risks and poorer diabetes quality of care which have been associated with substance abuse among patients with diabetes [3,4,5].

### 1.1. Why is the Review Important?

Shiri et al. [6] showed that opium abuse was a common problem among some people with diabetes, while Kim et al. [7] noted that substance abuse was on the increase globally and individuals who abuse substances are at increased risk of worse hypertension and diabetes outcomes. Therefore, it is essential to understand the association between unhealthy drug use and the clinical outcomes due to its public health significance including its impact on markers of cardiovascular diseases such as total cholesterol, high-density lipoprotein cholesterol, low-density lipoprotein cholesterol, and triglycerides [7]. Patients with diabetes and poor lipid profile are at increased risk of cardiovascular morbidity and mortality as these conditions are major risk factors for cardiovascular events. In fact, cardiovascular diseases are the leading cause of death globally, accounting for about 17.9 million deaths every year [8,9]. A range of factors including diabetes, lack of physical activities, poor dietary habits, and lifestyle choices have been implicated in the development of this condition [8,10]. There is also an increased risk of metabolic syndrome and diabetes in people who abuse substances due to nutritional deficiencies and increased cell damage [11]. According to Najafipour and Beik [12], while opium may temporarily increase blood lipids, its long term use may aggravate diabetes and dyslipidemia. In contrast, limiting substance use in patients with type 2 diabetes can improve clinical outcomes [13].

Despite these findings, it would appear that there is limited research on the impact of substance abuse including opium in people with diabetes and its role in lipid metabolism due in part to the nature of illicit drug use and the difficulty in conducting accurate assessment [13,14,15]. Patients with diabetes using opioids have been found to be less likely to receive recommended glycated hemoglobin and blood lipids testing and are more likely to experience diabetes-related hospitalizations than those not using opioids [13]. In addition, the effect of opium abuse on lipid profile in patients with diabetes remains inconsistent [12]. In our previous review [16], we found that substance abuse significantly lowered fasting blood glucose in patients with diabetes compared with control although differences were not significant with respect to postprandial blood glucose and glycated hemoglobin. Therefore, this review is a follow-up to our earlier review.

### 1.2. Aim

This was a systematic review and meta-analysis which evaluated the impact of opium abuse on lipid profile in patients with diabetes.

## 2. Method

This systematic review and meta-analysis was conducted in line with the preferred reporting items for systematic reviews and meta-analyses (PRISMA) guidelines [17].

### 2.1. Types of Studies

The studies included in this review were cross-sectional and case-control studies.

### 2.2. Types of Participants

All the participants included in this study were patients with diabetes (type 1 and type 2 diabetes).

### 2.3. Types of Interventions

People with diabetes who abused opium were compared with people with diabetes who did not abuse opium (control).

### 2.4. Outcome Measures

The following were the primary outcomes of interest: (1) Total cholesterol (TC) (mmol/L), (2) High-density lipoprotein (HDL) cholesterol (mmol/L), (3) Low-density lipoprotein (LDL) cholesterol (mmol/L), (4) Triglyceride (TG) (mmol/L). A secondary outcome of interest was body mass index (BMI) kg/m^2^.

### 2.5. Data Sources and Search Strategy

Three databases (Embase, PubMed, and PsycINFO) plus Google scholar were searched independently by two researchers (O.O., O.O.O.) for relevant articles from database inception to 18 July 2019. The search terms included synonyms and medical subject headings (MeSH) and these were combined using Boolean operators (AND/OR) in accordance with the Population, Intervention, Comparator, and Outcomes (PICO) framework (Table 1) [18]. The articles obtained from the different databases were exported to EndNote (Analytics, Philadelphia, PA, USA) for de-duplication.

### 2.6. Study Selection

**Figure 1:**
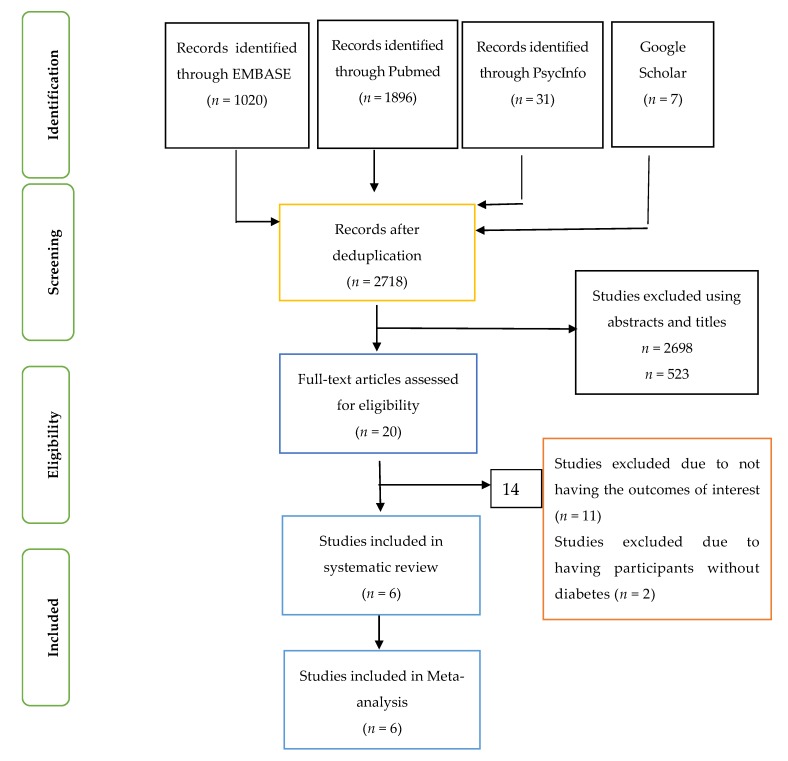
PRISMA FLOW CHART.

Articles were identified and screened using the abstracts and titles independently by two researchers (O.O., O.O.O.) and checked by two other researchers (X.-H.W., J.I.) (Figure 1). Based on the agreed inclusion and exclusion criteria by the four researchers, eligible articles were selected for inclusion in the systematic review and meta-analysis using the full text of the articles.

### 2.7. Inclusion and Exclusion Criteria

The following were the criteria for inclusion: Studies with (1) patients with type 1 and type 2 diabetes who were substance abusers, (2) case-control study, cross-sectional study, and retrospective cohort study, (3) outcomes of interest (lipid profile and body mass index).

Exclusion criteria were studies with: (1) Subjects with gestational diabetes, (2) outcomes without lipid profile, (3) participants abusing an unknown substance.

### 2.8. Rationale for the Studies Selected

The selection of studies was based on set criteria outlined above and only the studies that met these criteria were included in this review. Therefore, on this occasion, the studies that met the inclusion criteria were only those conducted in Iran. Other studies that were carried out in other countries around the world were also screened for eligibility and possible inclusion, but, were excluded due to not meeting the inclusion criteria. For example, Saunders et al. [19] study, which was conducted in the UK, was excluded from this review because the type of substance abused was not stated in the study. In addition, many other studies including Modzelewski et al. [20] study, conducted in USA, Lee et al. [21] study conducted in Australia, Saif-Ali et al. [22] study, carried out in Yemen, Isidro and Jorge [23] study, carried out in Spain, and Warner et al. [24] study, conducted in USA examined the effect of abuse of different substances in patients with diabetes. However, the outcome measures of cardio-metabolic significance in these studies were blood glucose parameters which are not the focus of the current review. None of these studies included lipid profile as measures of interest, hence, they were excluded from this review.

### 2.9. Data Extraction

Two researchers (O.O. and O.O.O.) extracted the data from the articles and these were cross-checked by two other researchers (X.-H.W. and J.I.).

### 2.10. Quality Evaluation

The Risk of Bias In Non-Randomised Studies of Interventions (ROBINS-I) assessment tool [25] was used to evaluate the quality of the studies included. These were in relation to the bias due to confounding, selection of participants into the study, classification of interventions, deviations from intended interventions, missing data, measurement of outcomes, selection of the reported result [25]. The risk of bias could be classified as low, moderate, serious or critical [25]. The process of the evaluation of the risk of bias was conducted by two researchers (O.O., O.O.O.) and cross-checked by the two other researchers (X.-H.W., J.I.). Only the data/information available in the studies selected were used to evaluate the different studies.

### 2.11. Statistical Analysis

The meta-analysis and sensitivity analysis were conducted using the Review Manager (RevMan 5.3 The Cochrane Collaboration, Copenhagen, Denmark) [26,27] and the random-effects models were used for the analysis due to the differences in the design of the studies included. Forest plots were used to illustrate the outcomes of the meta-analysis. A *p* value of <0.05 was considered as a measure of statistical significance for the overall effect of the intervention, whereas, the statistic *I*^2^ on a scale of 0–100% was used as the measure of heterogeneity across the studies. In addition, a *p* value of 0.1 was used to determine statistical significance of heterogeneity.

### 2.12. Data Inclusion Decisions

Data that were presented in mg/dL were converted to mmol/L when conducting the meta-analysis. The results for the Mohammadali et al. [28] and Rahimi et al. [29] studies which did not state whether values were a standard deviation or standard error of mean were recalculated using the means, sample sizes and the measures of dispersion [26] and the findings were comparable to standard deviation.

## 3. Results

Six studies published between 2004 and 2014 were selected for this systematic review and meta-analysis (Table 2 and Table 3). All the studies were conducted in Iran. Participants in five of the studies had type 2 diabetes while 91% in one study had people with type 2 diabetes. The type of substance abused was opium in all the studies. While four of the studies were cross-sectional studies, two were case-control studies.

### 3.1. Assessment of Risk of Bias of Included Studies

**Figure 2:**
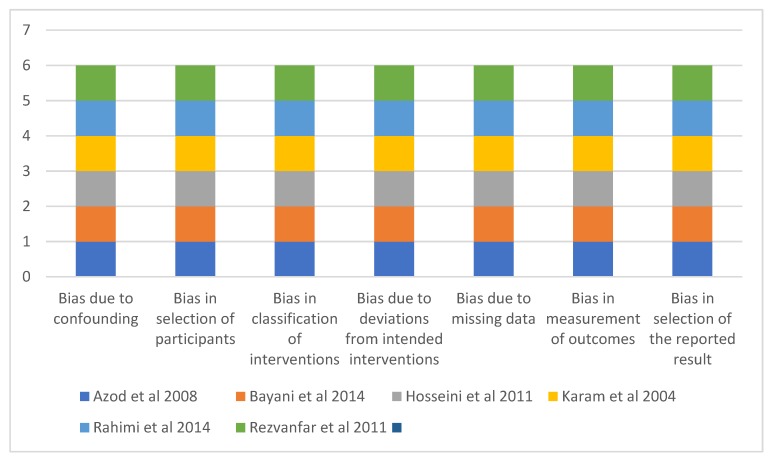
Showing a summary of the Risk of Bias (1 unit represents a low risk of bias while 2 represents a moderate risk of bias).

All the studies included in this review demonstrated a low risk of bias in all the domains assessed and had a low overall risk of bias (Figure 2).

### 3.2. Meta-Analysis of Data

**Figure 3:**
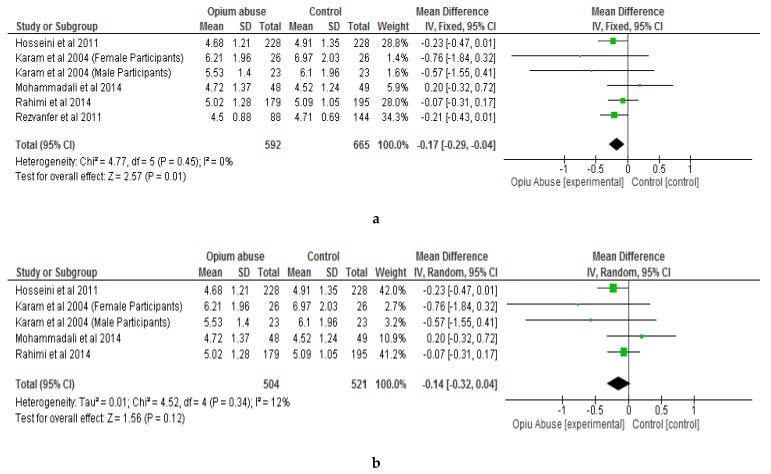
Showing the effect of opium abuse on Total Cholesterol (mmol/L). **a** Meta-analysis. **b** Sensitivity Analysis.

**Figure 4:**
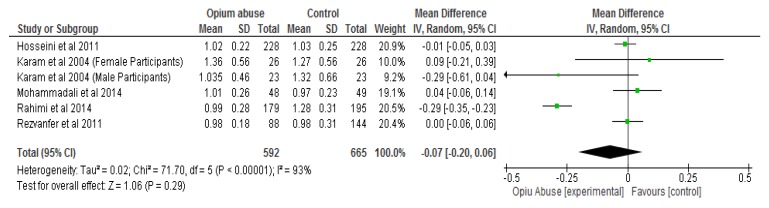
Showing the effect of opium abuse on high-density lipoprotein (HDL) cholesterol (mmol/L).

**Figure 5:**
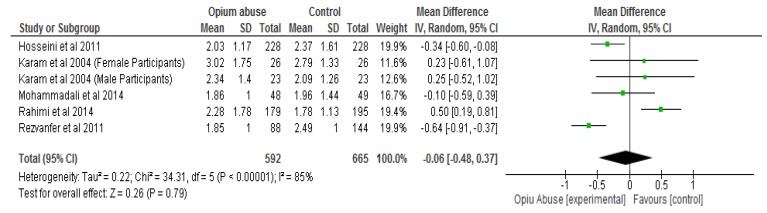
Showing the effect of opium abuse on Triglyceride (mmol/L).

**Figure 6:**
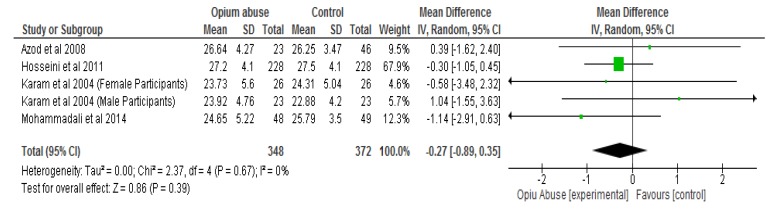
Showing the effect of opium abuse on Body Mass Index (kg/m^2^).

The results of the meta-analysis showed that opium abuse significantly (*P* = 0.01) lowered total cholesterol compared to control with a mean difference of −0.17 (95% CI, −0.29, −0.04) (Figure 3a). Following sensitivity analysis involving the removal of each study in turns, there were still significant differences (*P* < 0.05) between the two groups with respect to total cholesterol except in two studies [31,33] (Figure 3b) where differences were not statistically significant (*P* > 0.05). The differences with respect to HDL cholesterol, triglycerides and body mass index were not statistically significant (*P* > 0.05) between those who abused opium compared with control (Figure 4, Figure 5, Figure 6 respectively). The mean differences between the opium abuse and control groups were −0.07 (95% CI, −0.20, 0.06) for HDL cholesterol, −0.06 (95% CI, −0.48, 0.37) for triglyceride and −0.27 (95% CI, −0.89, 0.35) for body mass index.

**Figure 7:**
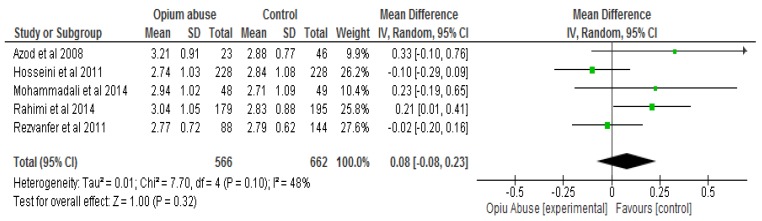
Showing the effect of opium abuse on low-density lipoprotein (LDL) cholesterol (mmol/L).

In relation to LDL cholesterol, the difference was also not statistically significant (*P* > 0.05) between the opium abuse and the control groups with a mean difference of 0.8 (95% CI, −0.08, 0.23) (Figure 7).

## 4. Discussion

The results of the systematic review and meta-analysis have shown that opium abuse has a significant effect (*P* < 0.05) on total cholesterol although the results of the sensitivity analysis did not demonstrate consistency in two of the studies [31,33] (Figure 3b). In addition, the effect of opium abuse on HDL cholesterol, triglycerides, LDL cholesterol, and body mass index was not significantly different (*P* > 0.05) compared with the control.

The findings of this review appear similar to the outcome of an earlier review [12] which evaluated the impact of opium on blood glucose and serum lipids in humans and animals. Although the study by Najafipour and Beik [12] was not a systematic review, it demonstrated that there were differences in the outcomes of the various studies on the impact of opium on lipid indices in humans. While some studies on healthy and patients with diabetes have shown no significant association between substance abuse and triglycerides, total cholesterol, LDL cholesterol, and HDL cholesterol, other studies have revealed that substance abuse has a harmful effect on one or more of lipid parameters [12].

In the current review, Karam et al. [32] found that opium abuse decreased HDL cholesterol and that total cholesterol tended to be lower in males who abused opium. On the other hand, Azod et al. [30] found no significant differences (*P* > 0.05) between the opium abuse group and the control group with respect to triglyceride, total cholesterol, LDL and HDL cholesterol. Rezvanfar et al. [33] noted that serum triglyceride in patients with diabetes who abused opium was significantly lower (*P* = 0.005) compared with control, while Mohammadali et al. [28] did not find significant difference (*P* > 0.05) between the opium abuse and control groups with respect to serum lipids.

The differences observed in the findings of some of the studies with respect to triglyceride, HDL and LDL cholesterol in this review could be due to differences in the sample size of the studies and the route of administration of the substances [34]. In addition, it could be due to the frequency of substance abused, the duration of drug dependency, socio-demographic characteristics and other comorbidities [11,12,16,18]. However, the significant decrease in total cholesterol in the participants who abused opium may be due to nutritional deficiencies. For example, Kouros et al. [35] concluded that low levels of lipids including cholesterol and triglycerides that are found in people who abuse substances are mainly due to drug-abuse associated malnutrition. There is evidence that substance abuse leads to loss of appetite in individuals who abuse drugs [34].

Asgary et al. [34] noted that nutritional habits, social status, and poor lifestyle may influence lipid profiles in individuals. The authors further reported that malnutrition in people who abuse substances could be a result of economic problems and/or individual choices. Furthermore, because opioids are metabolized mainly in the liver, chronic opium consumption may cause liver damage [12]. The liver plays a significant role in the metabolism of lipids, thus, chronic liver diseases may cause dysregulation of serum lipids [12]. In addition, opium suppresses appetite and reduces weight, therefore, the reduction in lipids may not be due to the direct effect of opium, instead it could be the result of weight loss or nutritional deficiency [12]. This position reaffirms the views expressed by Rahimi et al. [29] who observed that food regimen, malnutrition and physical activity which could be different in patients with diabetes who abuse opium compared with control are possible factors that could influence the level of cholesterol. Another possible factor that may affect the level of cholesterol in these subjects could be the impact of medications being administered to them [29]. However, in the current review, three studies [28,30,32] matched the patients with diabetes who abused opium with control in their medications. For example, Mohammadali et al. [28] noted that the participants in their study were on a range of medications including sulfonylurea agents, biguanides, insulin and statins and the two groups were matched in medications. Although the three other studies [29,31,33] did not discuss the medication history of the participants, the matching of the two groups in medications in the three other studies [28,30,32] would suggest a lesser role for medications in the current review with respect to the impact of opium abuse on cholesterol.

In relation to the BMI, the results which showed no significant difference (*P* > 0.05) between the group that abused opium and the control could be due to the fact that the two groups were matched for BMI in all the studies except in the Rahimi et al. [29] study where the mean BMI was significantly lower in the opium abuse group compared with the non-opium abuse group.

In terms of its public health significance, the findings of this review may be of relevance in the area of promoting adequate nutritional intake in patients with diabetes who abuse opium. In addition, the decrease in total cholesterol [36], underscores the potential impact of opium abuse on lipid metabolism in patients with diabetes.

### Limitations

All the studies included in this review were conducted in Iran, and this may affect its wider application. The Mohammadali et al. [28] and Rahimi et al. [29] studies did not state whether the data presented were expressed as means ± SD or means ± SEM. Although the SD was calculated based on the means, sample sizes and measures of dispersion and these were found comparable to the results presented, the non-inclusion of these information are limitations of those studies.

## 5. Conclusions

The findings of this systematic review and meta-analysis have shown that opium abuse significantly decreased (*P* < 0.05) total cholesterol in patients with diabetes. However, with respect to HDL cholesterol, triglycerides, BMI and LDL cholesterol, the differences were not statistically significant (*P* > 0.05) compared with control. This result may have public health significance in terms of ensuring the promotion of adequate nutritional intake in patients with diabetes who abuse opium. More studies are needed in order to fully understand the effect of opium abuse on lipid profile in patients with diabetes.

## Figures and Tables

**Table 1:** Search Terms and Search Strategy.

Patient/Population	Intervention	Comparator	Outcomes of Interest	Combining Search Terms
Patients with diabetes	Substance Abuse		Lipid Profile and Body Mass Index	
Type 2 diabetes OR type 1 diabetes OR diabetes complications OR diabetes mellitus, type 2 OR diabetes mellitus, type 1 OR diabetes mellitus	Substance-related disorders OR substance* OR marijuana abuse OR amphetamine-related disorders OR cocaine-related disorders OR opioid-related disorders OR opiate* OR opioid* OR heroin dependence		Body mass index OR BMI OR Total cholesterol OR High-density lipoprotein cholesterol OR HDL OR Low-density lipoprotein cholesterol OR LDL OR Triglycerides	Column 1 AND Column 2 AND Column 3

*(Truncation symbol).

**Table 2:** Characteristics of the articles included in this review (*N* = 6)

Study Reference	Country	Length of Study	Study Type/Design	Sample Size/Description	Age	Gender	Diabetes Type/Duration of Diabetes (YRS- Mean ± SD)	Type of Substance Abused
Azod et al. [30]	Iran	Not stated	Cross-sectional study	23 opium 46 non-opium The two groups were matched in age, BMI, duration of diabetes, cigarette smoking, medication, and education	Mean 60.52 ± 12.25 55.24 ± 10.92 years	All males	Type 2 DM Duration of diabetes recorded during the study, but not reported by authors.	Opium
Hosseini et al. [31]	Iran	2008–2010	Cross-sectional study	228 opium 228 non-opium The two groups were matched in age, BMI, sex and smoking status	Mean 58.9 (SD = 9.2 years	92% male	91% were type 2 DM Opium: 7.6 ± 7.1 non Opium: 8.2 ± 8.4	Opium
Karam et al. [32]	Iran	Not stated	Case-control study	23 male and 26 female opium 23 male and 26 female non-opium The two groups were matched in age, BMI, cigarette smoking and medication	35–65 years	53% female	Type 2 DM Duration of diabetes was not reported	Opium
Mohammadali et al. [28]	Iran	2006–2007	Cross-sectional study	48 opium users 49 non-opium users The two groups were matched in age, BMI, duration of diabetes and medication history	Mean 64 years	>60% female	Type 2 DM Opium: 11.31 ± 6.33 non Opium: 10.39 ± 7.91	Opium
Rahimi et al. [29]	Iran	Not stated	Cross-sectional study	179 opium users 195 non-opium users The two groups were not matched in age, BMI and cigarette smoking	Mean 53.5–58.2 years	Combined males and females	Type 2 DM Duration of diabetes was not reported	Opium
Rezvanfar et al. [33]	Iran	2009–2010	Case-control study	88 opium users 144 non-opium users The two groups were matched in age, BMI, duration of diabetes	Mean 55–57 years	All males	Type 2 DM Opium: 9.8 ± 6.4 non Opium: 7.8 ± 5.4	Opium

Abbreviation: BMI (Body Mass Index).

**Table 3:** Blood lipid Indicators of patients with diabetes based on their substance use status.

Study reference	Participants Studied	Body Mass Index (BMI) (kg/m^2^)	Total Cholesterol	High Density Lipoprotein (HDL)	Low Density Lipoprotein (LDL)	Triglycerides
Azod et al. [30]	**Substance abusers** Mean ± SD	26.64 ± 4.27	No data	34.98 mg/dL	123.96 ± 34.96 mg/dL	31.50 mg/dL
	**Non-substance abusers**	26.25 ± 3.47 *P* = 0.68	No data	35.01 mg/dL *P* = 0.99	111.24 ± 29.57 mg/dL *P* = 0.11	36.75 mg/dL *P* = 0.30
Hosseini et al. [31]	**Substance abusers** Mean ± SD	27.2 ± 4.1	180.96 ± 46.85 mg/dL	39.67 ± 8.80 mg/dL	105.82 ± 39.76 mg/dL	179.68 ± 103.15 mg/dL
	**Non-substance abusers**	27.5 ± 4.1 *P* = 0.391	189.85 ± 52.14 mg/dL *P* = 0.061	40.08 ± 9.70 mg/dL *P* = 0.640	109.63 ± 41.76 mg/dL *P* = 0.343	209.59 ± 142.12 mg/dL *P* = 0.012
Karam et al. [32] (for men)	**Substance abusers** Mean ± SEM	23.92 ± 0.68	5.53 ± 0.2 mmol/L	1.035 ± 0.066 mmol/L	No data	2.34 ± 0.2 mmol/L
	**Non-substance abusers**	22.88 ± 0.60 *P* = 0.2598	6.1 ± 0.28 mmol/L *P* = 0.098	1.32 ± 0.094 mmol/L *P* = 0.0376	No data	2.09 ± 0.18 mmol/L *P* = 0.3481
Karam et al. [32] (for women)	**Substance abusers** Mean ± SEM	23.73 ± 0.80	6.21 ± 0.28 mmol/L	1.36 ± 0.08 mmol/L	No data	3.02 ± 0.25 mmol/L
	**Non-substance abusers**	24.31 ± 0.72 *P* = 0.2200	6.97 ± 0.29 mmol/L *P* = 0.0711	1.27 ± 0.08 mmol/L *P* = 0.3483	No data	2.79 ± 0.19 mmol/L *P* = 0.5349
Mohammadali et al. [28]	**Substance abusers** Mean ± SD	24.65 ± 5.22	182.27 ± 53.23 mg/dL	39 ± 10.4 mg/dL	113.6 ± 39.25 mg/dL	164.46 ± 84.65 mg/dL
	**Non-substance abusers**	25.79 ± 3.5 *P* = 0.153	174.88 ± 47.89 mg/dL *P* = 0.307	37.47 ± 9.24 mg/dL *P* = 0.477	104.86 ± 42.1 mg/dL *P* = 0.171	173.49 ± 127.61 mg/dL *P* = 0.751
Rahimi et al. [29]	**Substance abusers** Mean ± SD	26.3 ± 5.6	194.1 ± 49.6 mg/dL	38.6 ± 10.9 mg/dL	117.6 ± 40.7 mg/dL	201.5 ± 157.3 mg/dL
	**Non-substance abusers**	27.7 ± 4.4 *P* = 0.009	196.9 ± 40.6 mg/dL *P* = 0.550	49.8 ± 12.2 mg/dL *P* < 0.001	109.4 ± 34.2 mg/dL *P* = 0.052	200.1 ± 99.8 mg/dL *P* = 0.910
Rezvanfar et al. [33]	**Substance abusers** Mean ± SD	No data	174 ± 34 mg/dL	38 ± 7 mg/dL	107 ± 28 mg/dL	164 ± 88 mg/dL
	**Non-substance abusers**	No data	182 ± 27 mg/dL *P* = 0.18	38 ± 12 mg/dL *P* = 0.90	108 ± 24 mg/dL *P* = 0.92	220 ± 86 mg/dL *P* = 0.005

Abbreviations: SD (Standard deviation), SEM (Standard Error of Mean).
